# Human Cholangiocytes Form a Polarized and Functional Bile Duct on Hollow Fiber Membranes

**DOI:** 10.3389/fbioe.2022.868857

**Published:** 2022-06-24

**Authors:** Zhenguo Wang, João Faria, Luc J. W. van der Laan, Louis C. Penning, Rosalinde Masereeuw, Bart Spee

**Affiliations:** ^1^ Division of Pharmacology, Department of Pharmaceutical Sciences, Faculty of Sciences, Utrecht Institute for Pharmaceutical Sciences, Utrecht University, Utrecht, Netherlands; ^2^ Department of Clinical Sciences, Faculty of Veterinary Medicine, Utrecht University, Utrecht, Netherlands; ^3^ Department of Surgery, Erasmus MC-University Medical Center, Rotterdam, Netherlands

**Keywords:** intrahepatic cholangiocyte organoids, cholangiocytes, bioengineered bile duct, hollow fiber membrane, monolayer, polarity, perfusable

## Abstract

Liver diseases affect hundreds of millions of people worldwide; most often the hepatocytes or cholangiocytes are damaged. Diseases of the biliary tract cause severe patient burden, and cholangiocytes, the cells lining the biliary tract, are sensitive to numerous drugs. Therefore, investigations into proper cholangiocyte functions are of utmost importance, which is restricted, *in vitro*, by the lack of primary human cholangiocytes allowing such screening. To investigate biliary function, including transepithelial transport, cholangiocytes must be cultured as three-dimensional (3D) ductular structures. We previously established murine intrahepatic cholangiocyte organoid-derived cholangiocyte-like cells (CLCs) and cultured them onto polyethersulfone hollow fiber membranes (HFMs) to generate 3D duct structures that resemble native bile ducts at the structural and functional level. Here, we established an efficient, stepwise method for directed differentiation of human intrahepatic cholangiocyte organoids (ICOs) into CLCs. Human ICO-derived CLCs showed key characteristics of cholangiocytes, such as the expression of structural and functional markers, formation of primary cilia, and P-glycoprotein-mediated transport in a polarized fashion. The organoid cultures exhibit farnesoid X receptor (FXR)-dependent functions that are vital to liver bile acid homeostasis *in vivo*. Furthermore, human ICO-derived CLCs cultured on HFMs in a differentiation medium form tubular architecture with some tight, confluent, and polarized monolayers that better mimic native bile duct characteristics than differentiated cultures in standard 2D or Matrigel-based 3D culture plates. Together, our optimized differentiation protocol to obtain CLC organoids, when applied on HFMs to form bioengineered bile ducts, will facilitate studying cholangiopathies and allow developing therapeutic strategies.

## Introduction

Cholangiocytes are epithelial cells that line both intra- and extra-hepatic bile ducts. They comprise only 3–5% of the total liver mass; however, their physiological function is important for regulating bile acids synthesis by the liver and their transport (Banales et al., 2019). Functional impairment of cholangiocytes plays an essential role in the development of various types of biliary disorders, collectively termed cholangiopathies. The physiology and pathophysiology of cholangiocytes and cholangiopathies are not fully elucidated as of yet (Lazaridis and Larusso, 2015) despite the large global burden and post-liver transplantation complications associated with cholangiopathies (Spada et al., 2009). This is mainly because research on the pathophysiology and the development of new treatment methods have been limited by the lack of robust platforms for disease modeling and drug screening studies, especially in men. Thus, novel *in vitro* models are needed to bring further progress in the field and mimic (intrahepatic) bile ducts in detail.

To create *in vitro* models that mimic cholangiocyte activity, several cell sources are available. Primary cholangiocytes can be freshly isolated from the human liver and cultured for a few weeks (Joplin, 1994). However, they tend to dedifferentiate due to the lack of a native environment. Another cell source frequently used is the cholangiocytes derived from human induced pluripotent stem cells (iPSCs). Advantages of iPSCs are the ability to undergo self-renewal and differentiation into cholangiocytes *in vitro*, but the efficiency for generating iPSC-derived cholangiocytes is low (Sampaziotis et al., 2017a). Moreover, this procedure is time-consuming, and their restricted differentiation and high cellular heterogeneity hamper their application. Hence, there is an obvious need for novel 3D *in vitro* models that more closely mimic the *in vivo* biliary trees in order to gain further insight into the physiology and pathophysiology of cholangiocytes.

Organoids offer ideal possibilities as a cell source in tissue engineering or regenerative medicine (Yin et al., 2016; Kim et al., 2020). Originally, liver organoids were generated from adult tissue progenitor/stem cells located in the intrahepatic bile ducts (Huch et al., 2015). *In vitro* culture of these progenitor/stem cells under Wnt/β-catenin-inducing conditions reveals organoids with an upregulation of the leucine-rich repeat-containing G protein-coupled receptor 5 (LGR5) stem cell marker (Planas-Paz et al., 2019). According to the new consensus on the nomenclature of the liver organoids originating from intrahepatic bile ducts, these are now termed intrahepatic cholangiocyte organoids (ICOs) (Marsee et al., 2021). The ICOs are highly proliferative and can be genetically stable and expanded for over several months *in vitro* (Huch et al., 2015). Furthermore, upon culturing with specific medium compositions, the ICOs have the capacity to differentiate into either hepatocyte (Schneeberger et al., 2020; Ye et al., 2020) or cholangiocyte (Chen et al., 2018) lineages. In addition, a novel method for the isolation and propagation of human cholangiocytes expanded from extrahepatic cholangiocyte organoids (ECOs) has been established. It should be noted that these ECOs were not cultured under Wnt/β-catenin-inducing conditions but under non-canonical Wnt-stimulated conditions. When implanted in mice, these human ECOs can reconstruct the common bile duct and replace its function in extrahepatic biliary injury (Sampaziotis et al., 2017b). Therefore, organoids can be an important cell source for regenerative medicine, but having a 3D platform that can replicate cholangiocyte (patho)physiology, including cholestasis, and allow drug screening is still not available.

Here, we reported a bioengineered 3D platform based on human ICOs differentiation toward CLCs. Furthermore, to acquire anatomical tubular architecture and an environment that mimics an intrahepatic bile duct, we cultured ICOs onto the polyethersulfone hollow fiber membrane (HFM). Genomic and functional analyses of CLCs demonstrated that this organoid culture manifested essential morphological and functional features of mature cholangiocytes, such as a polarized monolayer and tight barrier. Together, this bioengineered human bile duct more closely replicates the biological structure and function of native bile ducts and may be suitable for studying cholangiopathies and therapeutic interventions for these disorders.

## Materials and Methods

### ICO Establishment and Expansion

Healthy tissue samples of the human liver from two males and two females were obtained during liver transplantation at the Erasmus Medical Center in Rotterdam under the approval of the Medical Ethical Council (MEC-2014-060). The human liver-derived ICOs were isolated and cultured, as described previously (Broutier et al., 2016). Briefly, the liver biopsies were washed in cold phosphate-buffered saline (PBS) and minced into small pieces. Minced tissues were washed in cold DMEM GlutaMAX medium (Gibco, Thermo Fisher Scientific, Waltham, MA, United States) supplemented with 1% (v/v) fetal calf serum (FCS; Gibco) and 1% (v/v) penicillin/streptomycin (P/S; Gibco). This was followed by digestion with 0.125 mg/ml type II collagenase (Gibco) and 0.125 mg/ml dispase (Gibco) in the DMEM GlutaMAX medium supplemented with 0.1 mg/ml DNase I (Roche, Basel, Switzerland), 1% (v/v) FCS, and 1% (v/v) P/S at 37°C in a shaking water bath. Every 10–15 min, the supernatant was collected, and the fresh digestion-supplemented medium was added and allowed for digestion three times. Single cells were obtained by passing through a 70-µm filter, and the filtrate was washed in cold DMEM GlutaMAX medium (supplemented with 1% (v/v) FCS and 1% (v/v) P/S) and centrifuged (400 g for 5 min at 4°C). The cells were diluted in cold Matrigel (Corning, New York, NY, United States) and seeded in 50 µL droplets in 24-well plates (Corning). After gelatinization of the Matrigel, a culture medium was added, and cells were incubated at 37°C, 5% CO_2_ (v/v). The culture medium (expansion medium, EM) was based on the Advanced DMEM/F12 medium (Gibco) consisting of 1% (v/v) P/S, 1% (v/v) HEPES (10 mM; Gibco), and 1% (v/v) GlutaMax (Gibco). The EM was supplemented with 10% (v/v) R-Spondin-1–conditioned medium (the Rspon1-Fc-expressing cell line was a kind gift from Calvin J. Kuo), 2% (v/v) B27 supplement without vitamin A (Invitrogen, Carlsbad, CA, United States), 1% (v/v) N2 supplement (Invitrogen), 10 mM nicotinamide (Sigma-Aldrich, St. Louis, MO, United States), 1.25 mM N-acetylcysteine (NAC; Sigma-Aldrich), 100 ng/ml fibroblast growth factor 10 (FGF10; Peprotech, Rocky Hill, NJ, United States), 10 nM recombinant human (Leu15)-gastrin Ⅰ (GAS; Tocris Bioscience, Bristol, United Kingdom), 10 µM forskolin (Tocris Bioscience), 50 ng/ml epidermal growth factor (EGF; Peprotech), 25 ng/ml hepatocyte growth factor (HGF; Peprotech), and 5 µM A8301 (transforming growth factor *ß* inhibitor; Tocris Bioscience). Organoids were mechanically split weekly at 1:3–1:4 ratio, and the medium was changed every 2–3 days.

### Differentiation of Human ICOs to CLCs

ICOs were removed from Matrigel using cold Advanced DMEM/F12, mechanically dissociated into cell clusters using a pipette tip, and transferred to fresh Matrigel mixed with 1.2 mg/ml rat-tail type Ⅰ collagen (Merck Millipore, Darmstadt, Germany) at a ratio of 2:3. Cultures were subsequently incubated at 37°C for 2 hours in a culture incubator for the hydrogel to polymerize after which the EM medium was added. After 1 day, the medium was changed to defined medium (cholangiocyte differentiation medium, CDM) consisting of Advanced DMEM/F12 medium supplemented with 1% (v/v) P/S, 1% (v/v) HEPES, 1% (v/v) GlutaMax, 2% (v/v) B27 supplement without vitamin A, 1% (v/v) ITS Premix (contains insulin, human transferrin, and selenous acid; Corning), 1.25 mM NAC, 100 ng/ml FGF10, 10 nM GAS, 50 ng/ml EGF, 25 ng/ml HGF, and 5 µM A8301. Under differentiating conditions, cells were cultured for 1 week, and the medium (CDM) was refreshed every other day.

### RNA Isolation and RT-qPCR

The RNeasy Micro kit (QIAGEN, Hilden, Germany) was used to isolate RNA from liver organoids, M/C (Matrigel/collagen)- and HFM-cultured CLCs, following the manufacturer’s instructions. RNA concentration and purity were measured with the ND-1000 spectrophotometer (NanoDrop, Thermo Fisher Scientific). cDNA was prepared with the iScript cDNA synthesis kit, according to the manufacturer’s instructions (Bio-Rad, Hercules, California, United States). qPCR was performed to measure the relative gene expression using the SYBR Green method (Bio-Rad). Hypoxanthine phosphoribosyltransferase 1 (*HPRT1*) and ribosomal protein L19 (*RPL19*) were selected as stably expressed reference genes, and their average expression was used for normalization (Bustin et al., 2010). Details of all primers are described in Supplementary Table S1. Primary cells might lose some characteristics upon *in vitro* culturing. Therefore, we used the human gallbladder that contains mature cholangiocytes to compare the gene expression of markers in CLCs. Hence, we normalized the CLC gene expression to gallbladder samples for data analysis.

### Cholangiocyte Functional Studies

A rhodamine 123 (Sigma-Aldrich) transport assay was performed on day 4. Organoids were pretreated with DMSO or 10 µM verapamil [an inhibitor of the efflux pump P-glycoprotein also known as multidrug resistance protein 1 (MDR1); Sigma-Aldrich] and incubated at 37°C for 30 min. Subsequently, organoids were gently removed from Matrigel/collagen and resuspended in CDM containing 100 µM of rhodamine 123 and incubated at 37°C for 10 min. Then, organoids were centrifuged (400 g for 5 min at room temperature), and the supernatant was removed. Organoids were washed with warm Advanced DMEM/F12 and centrifuged again. Fluorescence (excitation wavelength: 470 nm; emission wavelength: 535 nm) was measured using the BZ-9000 microscope (Keyence, Osaka, Japan).

For the farnesoid X receptor (FXR) activity assay, CLCs were incubated in CDM consisting of 10 µM GW4064 (an agonist of FXR; Sigma-Aldrich) or DMSO (control) for 24 h. RNA was isolated and used to evaluate the gene expression of downstream signaling of FXR target genes (solute carrier family 51 subunit alpha and solute carrier family 51 subunit beta, *SLC51A* and *SLC51B*) in the CLCs.

### Culture of CLCs on the HFM

The polyethersulfone HFM (SENUO Filtration Technology, Tianjin, China) was double-coated to provide the cells with an extracellular matrix, as described previously (Chen et al., 2018). Briefly, HFM were cut into 3-cm pieces and sterilized in 70% (v/v) ethanol for 30 min. Next, the HFM was washed in PBS and precoated with filter-sterilized l-DOPA solution (2 mg/ml of 3,4-Dihydroxy-l-phenylalanine in 10 mM Tris buffer, pH 8.5) for 4 h at 37°C in an incubator, during which the fibers were turned 90° every hour. Subsequently, fibers were washed with PBS and put in rat-tail type I collagen solution (25 μg/ml in PBS) for another 2 h at 37°C in an incubator, during which the fibers were turned 90° every 30 min. The collagen solution was removed, and the fibers were washed with PBS prior to cell seeding. Single cells were prepared from liver organoids by in-gel trypsinization into single cells using TrypLE™ Express (Gibco) in a culture plate for 30–45 min at 37°C in an incubator. The single cells were collected and washed with Advanced DMEM/F12. Next, the double-coated HFMs were incubated with the cell suspension (5 × 10^5^ cells/3-cm fiber in 1.5-ml Eppendorf tubes) in 1 ml EM for 4–5 h at 37°C to allow cell adherence to the membrane. The tubes were turned 90° every hour. Finally, the cells covered HFM were transferred to 6-well plates and cultured in EM for 1–2 weeks for expansion and subsequently in CDM for 1 week for differentiation to create the bile duct tubules.

### Immunofluorescence Analysis

ICO-derived CLCs were removed from M/C using ice-cold cell recovery solution (Corning) and incubated on a horizontal shaker at 4°C (60 rpm) for 30–60 min until the 3D drops were dissolved. M/C- and HFM-cultured CLCs were washed with PBS and fixed with 4% (v/v) paraformaldehyde solution for 30 min at 4°C and permeabilized with 0.3% (v/v) Triton X-100 in PBS for 30 min at 4°C. To avoid non-specific antibody binding, the samples were incubated with block solution [2% (v/v) goat serum (Sigma-Aldrich), 2% (w/v) bovine serum albumin (Sigma-Aldrich), and 0.1% (v/v) Tween-20 in PBS (0.1% PBST)] for 1 h. Primary antibodies were diluted in block solution, and samples were incubated overnight at 4°C with shaking (60 rpm on a horizontal shaker). After being washed with 0.1% PBST three times for 20 min while shaking at 4°C (60 rpm on a horizontal shaker), the samples were incubated with secondary antibodies (5 µM Alexa Fluor 488 or 568; Life Technologies, Thermo Fisher Scientific) and with DAPI to stain nuclei (1:1,000; Sigma-Aldrich) overnight at 4°C while shaking (60 rpm on a horizontal shaker). Finally, tissues were washed three times for 20 min while shaking at 4°C (60 rpm on a horizontal shaker) and mounted on slides with ProLong Diamond Antifade mounting medium (Invitrogen). Images were acquired using a Leica TCS SP8 X imaging system. Antibody details for experiments are shown in Supplementary Table S2.

### Trans-Epithelial Barrier Function

To measure the permeability of the bioengineered bile duct, an inulin-fluorescein isothiocyanate (2–5 kDa; inulin-FITC, Sigma-Aldrich) leakage assay was performed. The bile duct fiber was connected to inlet and outlet blunt needles (18 g; 0.5-inch length; OctoInkjet, Hoyland, United Kingdom) assembled in a custom-made 3D-printed biocompatible polylactide chamber (Supplementary Figure S1). Bile ducts were perfused (2 ml/h) with 0.1 mg/ml inulin-FITC in HBSS buffer supplemented with 10 mM HEPES for 10 min at room temperature. Samples were taken from the outer compartment of the bile duct, and fluorescence was measured at an excitation wavelength of 475 nm and an emission wavelength of 500–550 nm by using a fluorometer (Promega; Madison, Wisconsin, United States).

### Statistical Analysis

**FIGURE 1:**
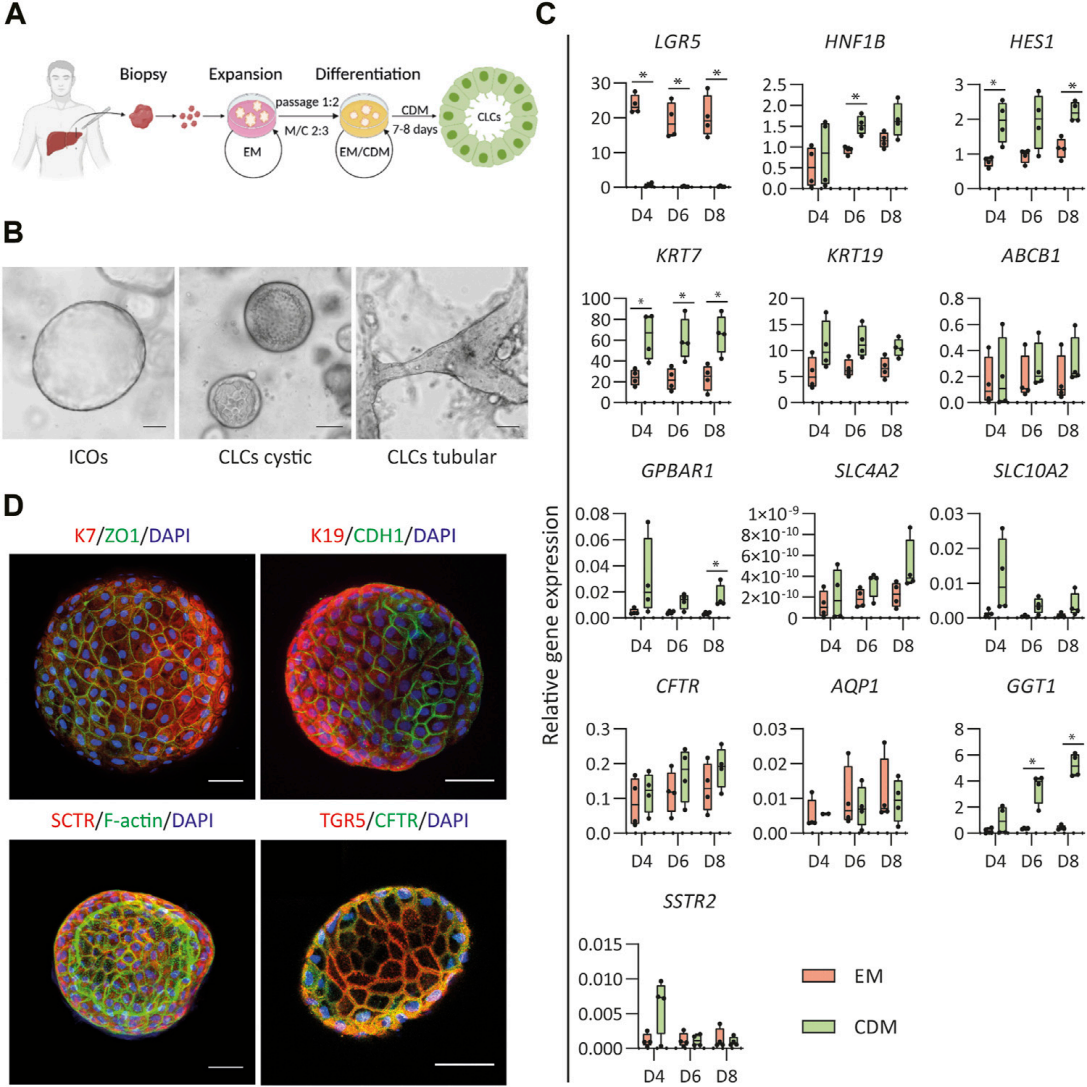
Differentiation of human intrahepatic cholangiocyte organoids (ICOs) toward cholangiocyte-like cells (CLCs). **(A)** Schematic overview of the protocol for differentiation of human ICOs to CLCs. EM, expansion medium; CDM, cholangiocyte differentiation medium; M/C, Matrigel/collagen type Ⅰ mixture. **(B)** ICOs in EM condition and cystic/tubular structures of CLCs formed in CDM. Left, ICOs; Middle, CLCs cystic; Right, CLCs tubular structure. Scale bar = 100 μm. **(C)** Gene expression analysis for ICOs under EM and CDM conditions. N = 4 independent donors for EM and CDM conditions at three time points (days 4, 6, and 8). Results are shown as fold change relative to the human gallbladder for cholangiocyte markers and the liver tissue for adult stem cell markers. Data are shown as box and whisker plots. Center line, median; box, interquartile; whiskers: minimum to maximum, show all points. Statistical differences between groups (EM and CDM) were determined by the two-tailed Mann–Whitney U test (see also Supplementary Table S3). *p < 0.05. **(D)** Immunofluorescence analysis of the CLCs expression of key cholangiocyte markers. Typically, in a well of a 96-well plate, about 50 organoids are cultured: keratin (K) 7 and K19, transport markers [secretin receptor (SCTR), G protein-coupled bile acid receptor 1 (GPBAR1, also known as TGR5), cystic fibrosis transmembrane conductance regulator (CFTR)], epithelial markers [cadherin 1 (CDH1), and tight junction protein 1 (ZO1)]. In addition, the F-actin is performed on the organoids’ apical surface facing the central lumen. Scale bar = 50 μm.

**FIGURE 2:**
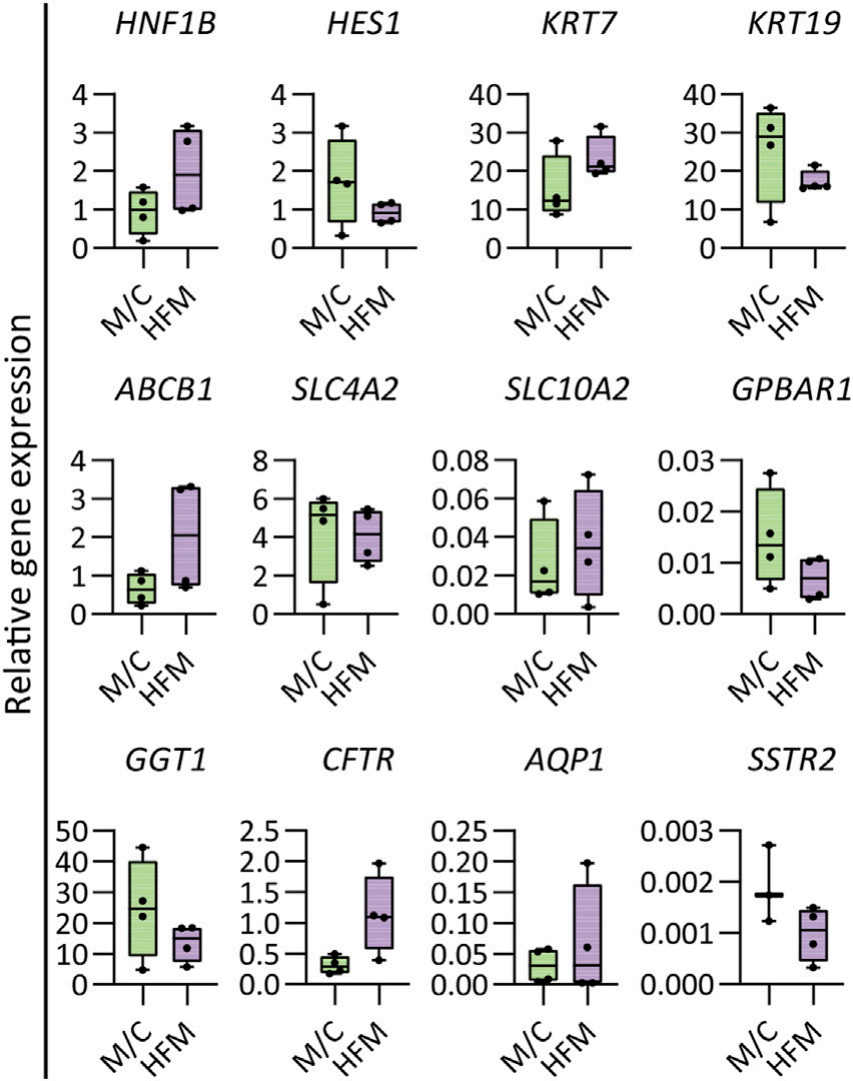
Gene expression in mature bile ducts. Gene expression analysis demonstrating the HFM culture maintained cholangiocyte characteristics and expressed some more mature cholangiocyte characteristics compared to M/C-cultured CLCs. Results are shown as fold change relative to the human gallbladder for cholangiocyte markers. *N* = 4 independent donors for M/C- and HFM-cultured CLCs in CDM conditions on day 7. Data are shown as box and whisker plots. Center line, median; box, interquartile; whiskers: minimum to maximum and show all points. Statistical differences between groups were analyzed using the two-tailed Mann–Whitney U test (see also Supplementary Table S4).

**FIGURE 3:**
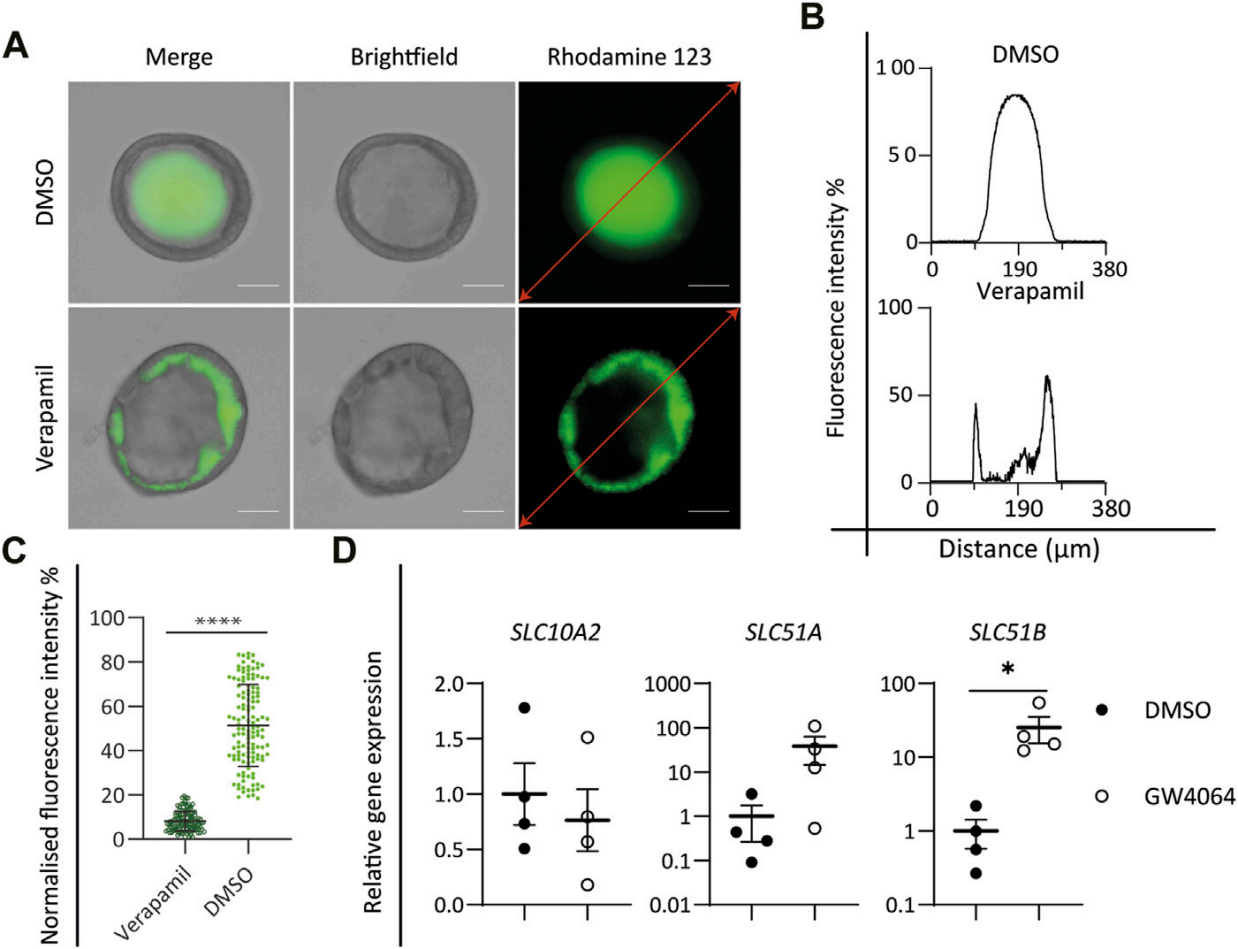
Functional characterization of cholangiocyte-like cells (CLCs). **(A–C)** Rhodamine 123 transport assay. **(A)** Brightfield and fluorescent images showing the P-glycoprotein transport function by active transport of rhodamine 123, DMSO as control, and verapamil as an inhibitor of P-glycoprotein function. Scale bar = 50 μm. Graphs depicting the fluorescence intensity along the red lines in the images in **(A)**. **(C)** Mean intraluminal fluorescence intensity normalized to background levels for cystic cholangiocyte. Data are shown as mean ± SD for each group. Statistical differences between groups were analyzed using the two-tailed Mann–Whitney U test; verapamil, *N* = 127; DMSO, N = 132; ****p < 0.0001. **(D)** FXR assay. Gene expression analysis showing the activation of FXR signaling downstream target genes, *SLC10A2, SLC51A*, and *SLC51B* in DMSO versus the FXR agonist (GW4064)-treated group. *N* = 4 independent donors for each group. Data are shown as mean ± SEM of four independent experiments for each group. Statistical differences between groups were determined using the paired *t*-test; DMSO, *N* = 4; GW4064, *N* = 4; *p < 0.05.

**FIGURE 4:**
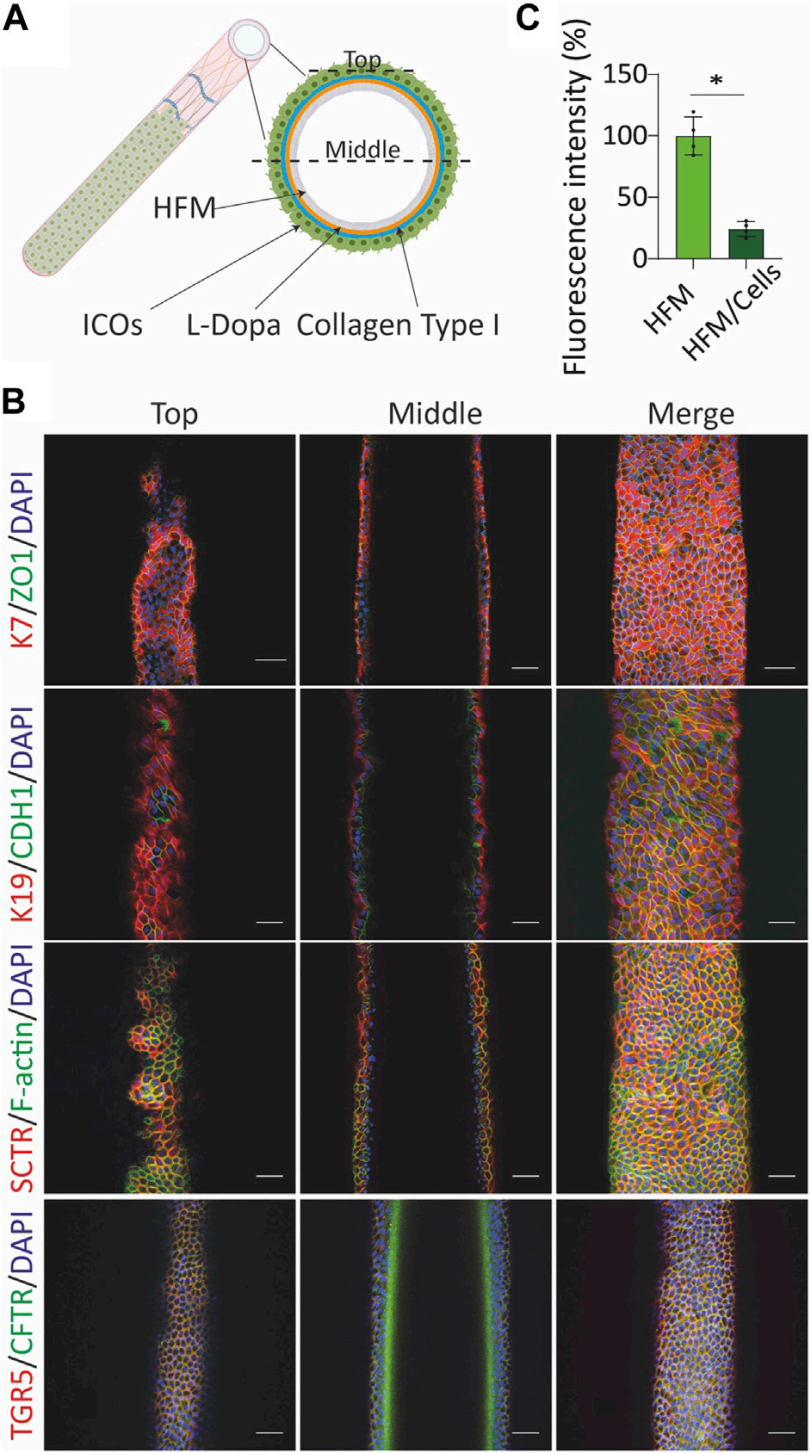
Culture of cholangiocyte-like cells (CLCs) on the extracellular matrix (ECM)-coated hollow fiber membrane (HFM) form tight and mature bile duct. **(A)** Illustrative image of HFM-cultured CLCs. **(B)** Bioengineered bile duct immunostaining. Immunofluorescence analysis of the bioengineered bile duct expression of key cholangiocyte markers [keratin (K) 7 and 19], transport markers [(secretin receptor (SCTR), G protein-coupled bile acid receptor 1 (GPBAR1, also known as TGR5), cystic fibrosis transmembrane conductance regulator (CFTR)], F-actin, epithelial markers [cadherin 1 (CDH1), and tight junction protein 1 (ZO1)]. Scale bar = 50 μm. **(C)** Trans-epithelial barrier function. The inulin-FITC leakage was assessed in the presence or absence of CLCs; data are shown as mean ± SD of four independent experiments for each group. Statistical differences between groups were analyzed using the two-tailed Mann–Whitney U test; HFM, *N* = 4; HFM/Cells, *N* = 4; *p < 0.05.

Statistical graphs were determined by GraphPad Prism 9 (GraphPad Software). RT-qPCR results’ data were presented as data points in box and whiskers (Min to Max; Figures 1C, 2) and the mean ± standard deviation (mean with SD; Figures 3C,D, 4C). The statistical test used was the two-tailed Mann–Whitney *U* test, and *p*-value details are described in the figure legends.

## Results

### *In Vitro* Generation of CLCs From ICOs

The generation of CLCs from human ICOs was based on our previous strategy for murine liver organoids (Chen et al., 2018) with some modifications to enhance further differentiation (Figure 1A). Compared to the study by Chen et al., we modified the medium and used a serum-free culture system to drive the human ICOs to CLCs. Details of expansion and differentiation medium are described in Supplementary Table S5. ICOs were expanded from the liver tissue biopsy, as previously described (Broutier et al., 2016), and seeded in a matrix that consisted of Matrigel and type Ⅰ collagen. After 8 days of cholangiocyte differentiation, organoids formed cystic and tubular structures whereas the organoids growing in expanding conditions remained cystic (Figure 1B). The cholangiocyte organoids showed an increase in wall thickness, and the central lumen became more visible. In addition, the CLCs demonstrated more clearly visible membrane junctions than the organoids cultured under expansion conditions.

Next, the efficiency of the differentiation was evaluated through gene expression analysis performed at three different time points (day 4, 6, and 8) in expanding (EM) and differentiating (CDM) conditions. EM analysis served as a control to assure differentiation was performed toward more mature cholangiocytes. The first indicator of differentiation was the decreased expression of the stem/progenitor cell marker *LGR5* in the CDM condition compared to EM (Figure 1C), which was decreased at all time points. The enhanced mRNA expression of the biliary markers hepatocyte nuclear factor-1β (*HNF1β*) and hairy and enhancer of split-1 (*HES1*) demonstrated a more efficient cholangiocytic phenotype. Under the CDM condition, *HNF1β* was increased on day 6, and *HES1* was increased on days 4 and 8. This differentiation was further corroborated by the increased mRNA expression of keratin 7 (*KRT7*) at all time points (days 4, 6, and 8). G protein-coupled bile acid receptor 1 (*GPBAR1*) on day 8 and gamma-glutamyl transferase 1 (*GGT1*) on days 6 and 8 were increased under differentiation conditions. No significant difference in *HNF1β* (days 4 and 8), *HES1* (day 6), keratin 19 (*KRT19*; days 4, 6, and 8), ATP binding cassette subfamily B member 1 (*ABCB1*; days 4, 6, and 8), *GPBAR1* (days 4 and 6), solute carrier family 4 membrane 2 (*SLC4A2*; days 4, 6, and 8), solute carrier family 10 membrane 2 (*SLC10A2*; days 4, 6, and 8), cystic fibrosis transmembrane conductance regulator (*CFTR*; days 4, 6, and 8), *GGT1* (day 4), and somatostatin receptor 2 (*SSTR2*; day 4) were found, although the mRNA expressions under CDM conditions were higher than EM. Only for the biliary markers, aquaporin 1 (*AQP1*; days 4, 6, and 8) and *SSTR2* (days 6 and 8), the mRNA expression was slightly lower under CDM conditions than EM. More details on the statistical analyses are presented in Supplementary Table S3.

To further verify the organoid differentiation to CLCs, immunofluorescence staining was performed (Figure 1D). Epithelial markers, tight junction protein 1 (TJP1, also known as ZO1), and E-cadherin 1 (CDH1), as well as cholangiocyte markers K7 and K19, were present in CLCs. More importantly, the CLCs expressed cholangiocyte-specific transporters, secretin receptor (SCTR), and TGR5 (also known as GPBAR1, encoded by *GPBAR1*) that play an important role in sensing and signaling and appear in mature cholangiocytes (Guo et al., 2016; Sato et al., 2018; Wang et al., 2021). In addition, the CLCs showed F-actin indicated by the staining for phalloidin, as a marker for the apical domains which demonstrates the CLCs developed apicobasal epithelial polarity, with the apical surface facing the lumen.

### Functional Characterization of CLCs

In the liver, cholangiocyte functions including the reabsorption and secretion of bile acids, water, and small molecules are performed by a series of transmembrane channel proteins. Cholangiocytes harbor a variety of transport channels, for instance, P-glycoprotein (also known as MDR1, encoded by *ABCB1*), a mediator of multidrug resistance expressed at the apical surface. To determine the functionality of *in vitro*-generated CLCs, the function of P-glycoprotein was investigated by incubating CLC organoids with the fluorescent dye rhodamine 123. Functional P-glycoprotein activity, located at the apical membrane, was confirmed by the accumulation of rhodamine 123 inside the lumen of CLC organoids. Moreover, blocking the transport activity with verapamil resulted in the accumulation of the fluorescent dye in the cytoplasm of organoid cells, verifying specific P-glycoprotein-mediated transport activity (Figures 3A–C).

Activation of the nuclear bile acid receptor farnesoid X receptor (FXR) leads to bile excretion whereas its inhibition causes bile accumulation or cholestasis (Shin and Wang, 2019). Bile acid excretion is performed by two transporters: one of ASBT located at the apical membrane of the cholangiocyte and the organic solute transporter α/β (OST α/β) (encoded by *SLC51A* and *SLC51B*, respectively) at the basolateral membrane. ASBT is responsible for the uptake of bile acids and can be downregulated by FXR activation. On the other hand, OST α/β are upregulated by FXR and support bile acid excretion. Incubation of the CLC organoids with the well-known FXR agonist GW4064 resulted in the downregulation of *SLC10A2* and in the upregulation of *SLC51A* and *SLC51B* (Figure 3D), indicating that the ICO-derived CLCs express functional FXR-mediated bile homeostasis.

### CLCs Form Tight and Mature Bile Ducts When Cultured on the HFM

The observed polarization and functionality of the organoids prompted us to create a HFM system amendable for transport studies. For this, single cells were acquired from trypsinized organoids and seeded on the HFM scaffold (Figure 4A). The cell-seeded constructs were cultured for 10–14 days under expanding conditions (EM) until a confluent monolayer was formed and subsequently switched to differentiating conditions (CDM) for seven more days. CLCs formed an epithelial barrier on the HFM, as confirmed by immunofluorescence staining of the tight junction proteins ZO1 and CDH1 (Figure 4B). Furthermore, the restricted inulin-FITC diffusion represented the bile duct barrier function (Figure 4C). The bile ducts show an inside-out polarization as staining for the tight junction protein and phalloidin demonstrated extraluminally expressed TJP1/ZO1 and F-actin, whereas the basolateral E-cadherin/CDH1 and SCTR appeared at the luminal side. In addition, staining for K7, K19, TGR5/GPBAR1, and CFTR confirmed that HFM-cultured CLCs also maintained cholangiocyte-specific markers while being cultured on HFM (Figure 4B).

The bioengineered bile ducts were characterized further by comparing gene expression profiles to organoids cultured in CDM on a culture plate (M/C). The results revealed that CLCs cultured on HFMs maintained a stable expression of key cholangiocyte markers (*HES1*, *KRT19*, *SLC4A2*, *GPBAR1*, *GGT1*, and *SSTR2*). Interestingly, the cholangiocyte markers *HNF1β, KRT7*, *ABCB1*, *SLC10A2*, *CFTR*, and *AQP1* showed a trend toward upregulation in CLCs cultured on HFMs as compared to plates cultured under differentiating conditions, suggesting that some cholangiocyte markers show a more mature phenotype in these tubular structures (Figure 2). The details of the statistical analyses are presented in Supplementary Table S4.

## Discussion

The current study and application show that ICOs can be differentiated into more mature and functional cholangiocytes. In addition to the well-described differentiation toward hepatocyte-like cells, these data clearly confirm the bipotentiality of the human ICOs (Kruitwagen et al., 2020; Schneeberger et al., 2020; Ye et al., 2020; Bouwmeester et al., 2021). Moreover, this study provides a robust protocol to reproduce functional cholangiocytes from human ICOs and form a polarized and functional bioengineered human bile duct on the polyethersulfone HFM, making this bioengineered system compliable for transport studies, disease modeling, and drug development.

To develop an efficient cholangiocyte differentiation method, Chen et al. (2018) demonstrated that gastrin, N-acetylcysteine, and FGF10 maturated murine ICOs toward cholangiocyte-like cells, and EGF played an important role in directing cholangiocyte differentiation of liver progenitor cells (Kitade et al., 2013). In addition, Tanimizu et al. (2007) reported that a combination of EGF and HGF induced cyst formation for liver progenitor cells more than either EGF or HGF alone. Matrigel matrix is effective for the liver organoid culturing and differentiation (Nuciforo and Heim, 2021), but the combined Matrigel/collagen (M/C) better supports cyst formation of progenitor cells and cholangiocyte function (Tanimizu et al., 2007). Here, we showed that a combination of these compounds and hydrogel mixture, in combination with serum-free conditions, is sufficient to promote ICO differentiation into cholangiocyte-like cells with high efficiency. Importantly, our data show the CLCs expressed mature cholangiocyte markers and obtained several vital functions of cholangiocytes, including P-glycoprotein-mediated transport and FXR-dependent regulation. Most *in vitro* studies of cholangiopathies that have been reported relied on 3D organoid modeling (Artegiani et al., 2019; Ye et al., 2020; Zhao et al., 2020). Primary cell or stem-cell derived cholangiocytes in 3D organoid cultures better mimic the *in vivo* environment and differentiate well and can be used for barrier functions and transport measurements. However, 3D organoids are highly heterogeneous in shape and size, and their location is difficult to control in hydrogels. As the apical surface is located at the inside of the organoids, studying transepithelial transport is also extremely difficult. The device presented here represents a bile duct-on-chip platform that can build an actual tissue structure with cholangiocyte function and reduced variability and offers a novel tool to model and study cholangiopathies (Chen et al., 2018; Du et al., 2020).

Recently, synthetic and natural materials have been used to construct bioengineered bile ducts for transplantation purposes (Wang et al., 2021). Sampaziotis et al. (2017b) described collagen scaffolds populated by human extrahepatic organoids (ECOs) that formed a bioengineered bile duct, which were able to replace the native mouse extrahepatic bile duct. These results provided potential clinical applications for bioengineered bile duct transplantation. However, natural materials as scaffolds can easily collapse and degrade when transplanted. In addition, the ECOs used to create these bioengineered bile ducts are quite distinct to ICOs and show region-specific differentiation potential (Verstegen et al., 2020). Synthetic materials have also been used which have the main benefit of being durable and better chemically defined (Du et al., 2020). Du et al. (2022) reported polydimethylsiloxane (PDMS) support with a collagen scaffold to construct murine bile ducts, which are polarized and can be used to mimic barrier function (Du et al., 2020). However, these microfluidic devices were difficult to apply for transepithelial transport studies as the basal surface of the bioengineered bile duct attached to the collagen gel will hamper the diffusion speed, and/or compounds can get trapped in the collagen. In addition, even if the bioengineered bile duct consists of PDMS scaffolds, the inner channel formed with collagen and degradability of collagen (Meyer, 2019) could influence the bioengineered bile duct stability (Du et al., 2020). Therefore, alternative scaffold materials should be investigated and applied in *in vitro* studies or for clinical application. The polyethersulfone HFM used in the current study possesses biocompatibility and hemocompatibility, as well as excellent mechanical properties and ease of application in different types of organ biofabrication (Jansen et al., 2015; Jochems et al., 2019). In addition, we previously reported the use of HFM to model (murine) the liver (Chen et al., 2018). Due to the low cell adhesion capability of HFM scaffolds, the modified membrane using a double coating of L-3,4-dihydroxydiphenylalanine (l-DOPA) and collagen Ⅳ (Oo et al., 2011), the extracellular matrix coatings that promote formation and maintenance of epithelial monolayers (Zhang et al., 2009; Ni et al., 2011). To develop the murine bioengineered bile duct, Chen et al. (2018) described HFM coated with l-DOPA and collagen Ⅰ. This coating contributed to liver organoid-derived CLC monolayer formation and allowed polarized bile acid transport activity (Chen et al., 2018). Even though Chen et al. (2018) and Du et al. (2020) established the murine bioengineered bile ducts to study bile acid transport and disease modeling, respectively, the human bioengineered bile ducts are yet to be established for translational research.

In this study, we showed the combination of a scaffold of a double-coated biofunctionalized HFM together with CLCs organized into a human bioengineered bile duct. The generated human bioengineered bile duct recapitulated characteristics of bile ducts *in vivo*, including the 3D architecture. Furthermore, we demonstrated that ICO-derived CLCs polarized on the HFM and produced a confluent and tight monolayer with a barrier function. Even though the apical-basolateral polarity is opposite to the native bile duct and therefore not suitable for transplantation, it can still be applied in studying transepithelial transport or in drug screening. The opposite polarity of bioreactor devices has been applied earlier in drug screening and transport studies using a murine bile duct (Chen et al., 2018), as well as a bioengineered kidney (Jansen et al., 2015) and intestinal tubules (Jochems et al., 2019). In native bile ducts, the cholangiocytes lining the intrahepatic biliary tree display phenotypical heterogeneity (Banales et al., 2019). The large, but not small cholangiocytes, expressed the secretin and somatostatin receptors (Han et al., 2013). Importantly, our bioengineered bile ducts showed expressions of SCTR and *SSTR2*, which indicates that they have large bile duct characteristics. Cholangiocytes also expressed ASBT, which mediates bile acid uptake (Claro Da Silva et al., 2013), and TGR5, which plays an important role in bile acid signaling (Keitel and Haussinger, 2013; Masyuk et al., 2013). The cholangiocyte’s primary cilia are also different between small and large cholangiocytes (Huang et al., 2006). Primary cilia provide mechanosensory, chemosensory, and osmosensory functions and can detect changes in bile flow (Masyuk et al., 2006; Mansini et al., 2018). To identify the primary cilia presence, we selected *a*-tubulin as an indicator for immunostaining. We could confirm primary cilia presence; however, the organelles were small compared to a kidney cell line that demonstrated to have long primary cilia on each cell (Supplementary Figure S2A) and more infrequently. Recently, Ogawa et al. (2021) reported that human pluripotent stem cells induced the CFTR-positive cholangiocyte population that could generate functional ciliated cholangiocytes. Some of the described culture conditions in this article could benefit by helping with a more homogenous cilia formation in CLCs derived from ICOs. Overall, CLCs cultured on the HFM appear very similar to cholangiocytes *in vivo*, and their polarization allows cross-epithelial transport studies.

In conclusion, this study shows a robust methodology to differentiate ICOs toward the cholangiocytic lineage and provides a novel method to generate human bioengineered bile ducts. This methodology provides a novel *in vitro* model for studying bile duct function and will aid in drug development for cholangiopathies.

## Data Availability

The original contributions presented in the study are included in the article/Supplementary Material; further inquiries can be directed to the corresponding authors.
