# Supplementary material for: Human Cholangiocytes Form a Polarized and Functional Bile Duct on Hollow Fiber Membranes
Source: Front Bioeng Biotechnol. 2022 Jun 24;10:868857. doi: 10.3389/fbioe.2022.868857 (PMC9263983; doi:10.3389/fbioe.2022.868857)

Supplementary Material

1 Supplementary Tables

Supplementary Table 1. List of Primers used in RT-qPCR analysis

Gene	Forward primer	Reverse primer
RPL19	ATGAGTATGCTCAGGCTTCAG	GATCAGCCCATCTTTGATGAG
HPRT1	TATTGTAATGACCAGTCAACAG	GGTCCTTTTCACCAGCAAG
LGR5	GCAGTGTTCACCTTCCC	GGTCCACACTCCAATTCTG
HNF1B	ATGATCAAGGGTTACATGCAG	GTCTGGTTGAATTGTCGGAG
HES1	ACGTGCGAGGGCGTTAATAC	ATTGATCTGGGTCATGCAGTTG
KRT7	GGACATCGAGATCGCCACCT	ACCGCCACTGCTACTGCCA
KRT19	CTTCCGAACCAAGTTTGAGAC	AGCGTACTGATTTCCTCCTC
ABCB1	AATGATGCTGCTCAAGTTAAAGGG	TCAGTAGCGATCTTCCCAGAACC
SLC4A2	ACTACCTGAGTGACTTCCGA	TGCTACAGAACGAGAAGAAGG
SLC10A2	GTCTACCCTGGTACAGGTGC	GCGGGAAGGTGAATACGACA
GPBAR1	CATTGCCCACATTGCCAG	GAGCCAAGTAGACGAGGAG
GGT1	CCTCAAAGGGTACAACTTCTC	TTGTAGTAGGAGATCGGGTG
CFTR	TTCTGGGAGGAGGGATTTGG	GTGAGAAATTACTGAAGAAGAGGC
AQP1	CACCTCCTGGCTATTGACTACAC	ATCCAGTGGTTGCTGAAGTTGTG
SSTR2	GCAGTCCTCACATTCATCT	TGGTCTTCATCTTGGCATAG
SLC51A	TTGTTCGCCTCCCTATTCC	TTGTGGTCTTTCCTTCGGT

SLC51B	TGTGGTGGTCATTATAAGCATGG	TCTTAGGTTGTTTAGGCTGTTGTG

Supplementary Table 2. List of Antibodies used in immunofluorescent analysis

Antibody	Species	Supplier	Cat. No	Dilution
K7	Mouse	Dako	M7018	1:50
K19	Rabbit	Abcam	ab76539	1:150
ZO1	Rabbit	Invitrogen	40-2300	1:250
E-cadherin	Mouse	BD Bioscience	610181	1:100
SCTR	Rabbit	Atlas	HPA007269	1:200
GPBAR1	Rabbit	Atlas	HPA007269	1:200
CFTR	Mouse	Abcam	ab2784	1:200
α-tubulin	Mouse	Sigma-Aldrich	T6793	1:200
Phalloidin Alexa 488		Thermo Fisher	A-12379	1:200
Anti-mouse Alexa 488	Goat	Thermo Fisher	A-11029	1:200
Anti-Rabbit Alexa 488	Goat	Thermo Fisher	A-11034	1:200
Anti-mouse Alexa 568	Goat	Thermo Fisher	A-11004	1:200
Anti-Rabbit Alexa 568	Goat	Thermo Fisher	A-11036	1:200

Supplementary Table 3. Statistical analysis of the gene expression comparison between EM and CDM culture conditions

Gene name	Days	EM (n = 4) Median (Range)	CDM (n = 4) Median (Range)	Two tailed P value
LGR5	Day 4	23.0 (21.7 - 27.6)	0.51 (0.18 - 1.33)	0.0286
	Day 6	18.2 (14.8 - 25.5)	0.10 (0.09 - 0.44)	0.0286
	Day 8	19.1 (14.4 - 28.5)	0.11 (0.08 - 0.51)	0.0286
HNF1B	Day 4	0.51 (0.04 - 1.04)	0.85 (0.07 - 1.60)	0.4857
	Day 6	0.95 (0.79 - 1.00)	1.51 (1.27 - 1.80)	0.0286
	Day 8	1.15 (0.96 - 1.39)	1.61 (1.18 - 2.18)	0.1143
HES1	Day 4	0.81 (0.59 - 0.90)	1.97 (1.20 - 2.55)	0.0286
	Day 6	1.01 (0.66 - 1.09)	2.01 (0.94 - 2.81)	0.2000
	Day 8	1.16 (0.80 - 1.52)	2.18 (1.97 - 2.53)	0.0286
KRT7	Day 4	2.53 (15.25 - 32.79)	67.1 (38.3 - 82.8)	0.0286
	Day 6	21.5 (11.01 - 35.1)	57.3 (39.2 - 88.2)	0.0286
	Day 8	25.3 (7.9 - 37.2)	66.3 (42.2 - 88.0)	0.0286
KRT19	Day 4	4.90 (2.90 -9.6)	9.6 (6.9 - 17.3)	0.1143
	Day 6	6.18 (5.05 - 8.93)	11.1 (8.7 -15.6)	0.0571
	Day 8	6.57 (4.43 - 9.23)	10.4 (8.52 - 12.7)	0.0571
ABCB1	Day 4	0.086 (0.008 - 0.424)	0.109 (0.006 - 0.603)	>0.9999
	Day 6	0.11 (0.07 - 0.45)	0.20 (0.15 - 0.54)	0.2000
	Day 8	0.10 (0.04 - 0.45)	0.22 (0.20 - 0.59)	0.2000

GPBAR1	Day 4	0.0037 (0.0034 - 0.0081)	0.020 (0.005 - 0.074)	0.0571
	Day 6	0.0042 (0.0031 - 0.0053)	0.014 (0.005 - 0.018)	0.1143
	Day 8	0.0035 (0.0025 - 0.0046)	0.012 (0.011 - 0.029)	0.0286
SLC4A2	Day 4	$1.0085e^{-10}$ ($7.149e^{-12}$ -$3.012e^{-10}$)	$1.66325e^{-10}$ ($1.351e^{-11}$ - $5.146e^{-10}$)	0.6857
	Day 6	$1.78e^{-10}$ ($1.094e^{-10}$ - $2.933e^{-10}$)	$3.811e^{-10}$ ($1.4e^{-10}$ - $4.123e^{-10}$)	0.1143
	Day 8	$2.2785e^{-10}$ ($8.888e^{-11}$ - $3.492e^{-10}$)	$3.832e^{-10}$ ($3.335e^{-10}$ - $8.658e^{-10}$)	0.0571
SLC10A2	Day 4	0.0012 (0.0003 - 0.0027)	0.0089 (0.0033 -0.0258)	0.0571
	Day 6	0.0003 (0.0003 - 0.0009)	0.0034 (0.0008 - 0.00623)	0.1143
	Day 8	0.0004 (0.0002 - 0.0016)	0.0024 (0.0011 - 0.0087)	0.0571
CFTR	Day 4	0.082 (0.024 - 0.166)	0.123 (0.042 - 0.179)	0.4857
	Day 6	0.118 (0.043 - 0.193)	0.184 (0.067 - 0.241)	0.3429
	Day 8	0.128 (0.052 - 0.217)	0.192 (0.113 - 0.256)	0.3429
AQP1	Day 4	0.0030 (0.0028 - 0.0118)	0.0055 (0.0055 - 0.0056)	0.5333
	Day 6	0.0064 (0.0035 - 0.0230)	0.0070 (0.0008 - 0.0151)	0.8857
	Day 8	00071 (0.0061 - 0.0261)	0.0094 (0.0019 - 0.0164)	>0.9999
GGT1	Day 4	0.161 (0.008 - 0.409)	0.901 (0.070 - 2.091)	0.3429
	Day 6	0.353 (0.305 - 0.384)	3.97 (1.73 - 4.22)	0.0286
	Day 8	0.38 (0.28 - 0.67)	5.15 (4.40 - 6.13)	0.0286
SSTR2	Day 4	0.0009 (0.0002 - 0.0025)	0.0073 (0.0003 - 0.0005)	0.2000

	Day 6	0.0009 (0.0002 - 0.003)	0.0011 (0.0005 - 0.0021)	>0.9999
	Day 8	0.0007 (0.0005 - 0.0035)	0.0008 (0.0005 - 0.0019)	0.8857

Supplementary Table 4. Statistical analysis of the gene expression comparison between M/C and HFM

Gene name	M/C (n = 4) Median (Range)	HFM (n = 4) Median (Range)	Two tailed P value
HNF1B	0.99 (0.19 - 1.58)	1.90 (0.98 - 3.17)	0.3429
HES1	1.71 (0.33 - 3.17)	0.91 (0.65 - 1.17)	0.3429
KRT7	12.3 (8.8 - 27.8)	21.2 (19.4 - 31.6)	0.2000
KRT19	29.0 (6.7 - 36.5)	16.1 (15.6 - 21.5)	0.3429
ABCB1	0.64 (0.22 - 1.12)	2.052 (0.708 - 3.320)	0.3429
GPBAR1	0.0134 (0.0050 - 0.0275)	0.0070 (0.0029 - 0.0109)	0.1143
SLC4A2	5.17 (0.50 - 5.997)	4.147 (2.53 - 5.47)	0.6857
SLC10A2	0.0168 (0.0103 - 0.0585)	0.0341 (0.0034 - 0.0724)	0.6857
CFTR	0.284 (0.170 - 0.491)	1.10 (0.39 - 1.97)	0.0571
AQP1	0.0310 (0.0049 - 0.0573)	0.0315 (0.0026 - 0.1974)	>0.9999
GGT1	24.7 (4.7 - 44.5)	15.0 (5.7 - 18.5)	0.3429
SSTR2	0.0017 (0.0012 - 0.0027)	0.0011 (0.0003 - 0.0015)	0.2286

Supplementary Table 5.

Comparison between murine and human ICOs differentiation towards CLCs

Species	Stage specific differentiation	Culture conditions		
		**Medium**	**3D Plate Scaffold**	**Bioengineered bile duct**
Murine	ICOs Expansion	Advanced DMEM/F12 medium<br>1% (v/v) Penicillin/Streptomycin<br>1% (v/v) HEPES (10 Mm)<br>1% (v/v) GlutaMax<br>5% (v/v) Rspondin-1 conditioned medium<br>1% (v/v) B27 supplement without vitamin A<br>1% (v/v) N2 supplement<br>10 mM Nicotinamide<br>1.25 mM n-acetylcysteine<br>100 ng/mL FGF10<br>10 nM gastrin<br>10 µM Forskolin<br>50 ng/mL EGF<br>50 ng/mL HGF<br>5 µM A830	Matrigel	
	ICOs → CP	CEM conditions:<br>Advanced DMEM/F12 medium<br>1% (v/v) Penicillin/Streptomycin	Matrigel/Collagen<br>1.2 mg/mL rat-tail type I collagen at a ratio of 2:3	

		1% (v/v) HEPES (10 Mm)<br>1% (v/v) GlutaMax<br>10% (v/v) FBS<br>1% (v/v) Non-essential amino acids<br>50 ng/mL EGH<br>50 ng/mL HGF<br>0.1 µM dexamethasone		
	CP → CLCs	CEM supplemented with 1.25 mM n-acetylcysteine and 10 nM gastrin	Matrigel/Collagen<br>1.2 mg/mL rat-tail type I collagen at a ratio of 2:3	Double precoated HFM:<br>L-DOPA solution (2 mg/mL<br>Rat-tail type I collagen solution (25 µg/mL in PBS)
Human	ICOs Expansion	Advanced DMEM/F12 medium<br>1% (v/v) Penicillin/Streptomycin<br>1% (v/v) HEPES (10 Mm)<br>1% (v/v) GlutaMax<br>10% (v/v) Rspondin-1 conditioned medium<br>2% (v/v) B27 supplement without vitamin A<br>1% (v/v) N2 supplement<br>10 mM Nicotinamide<br>1.25 mM n-acetylcysteine<br>100 ng/mL FGF10<br>10 nM recombinant human (Leu15)-gastrin I<br>10 µM Forskolin	Matrigel	Double precoated HFM:<br>L-DOPA solution (2 mg/mL<br>Rat-tail type I collagen solution (25 µg/mL in PBS)

		50 ng/mL EGF<br>25 ng/mL HGF<br>5 µM A830		
	ICOs → CLCs	CDM conditions:<br>Advanced DMEM/F12 medium<br>1% (v/v) Penicillin/Streptomycin<br>1% (v/v) HEPES (10 Mm)<br>1% (v/v) GlutaMax<br>2% (v/v) B27 supplement without vitamin A<br>1% (v/v) ITS Premix (contains insulin, human transferrin and selenous acid)<br>1.25 mM n-acetylcysteine<br>100 ng/mL FGF10<br>10 nM gastrin<br>50 ng/mL EGF<br>25 ng/mL HGF<br>5 µM A830	Matrigel/Collagen<br>1.2 mg/mL rat-tail type I collagen at a ratio of 2:3	Double precoated HFM:<br>L-DOPA solution (2 mg/mL<br>Rat-tail type I collagen solution (25 µg/mL in PBS)
FBS: Fetal Bovine Serum; EGF: Epidermal Growth Factor; HGF: Hepatocyte Growth Factor; FGF 10: Fibroblast Growth Factor 10.				

2 Supplementary Figures

2.1 Supplementary Figures

Supplementary Figure 1.

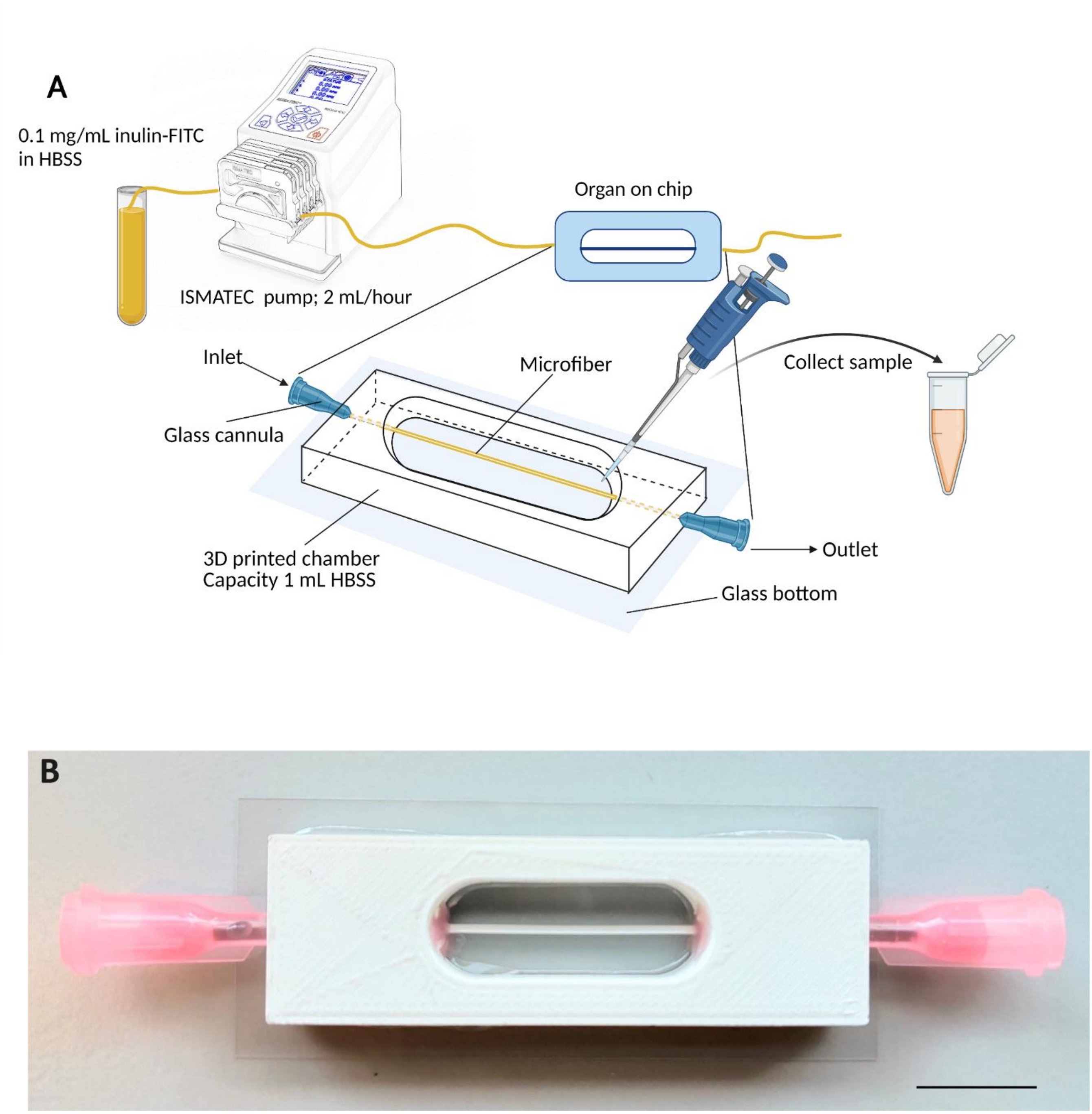
A
0.1 mg/mL inulin-FITC
in HBSS
Organ on chip
ISMATEC pump; 2 mL/hour
Inlet
Microfiber
Collect sample
Glass cannula
Outlet
3D printed chamber
Capacity 1 mL HBSS
Glass bottom
B

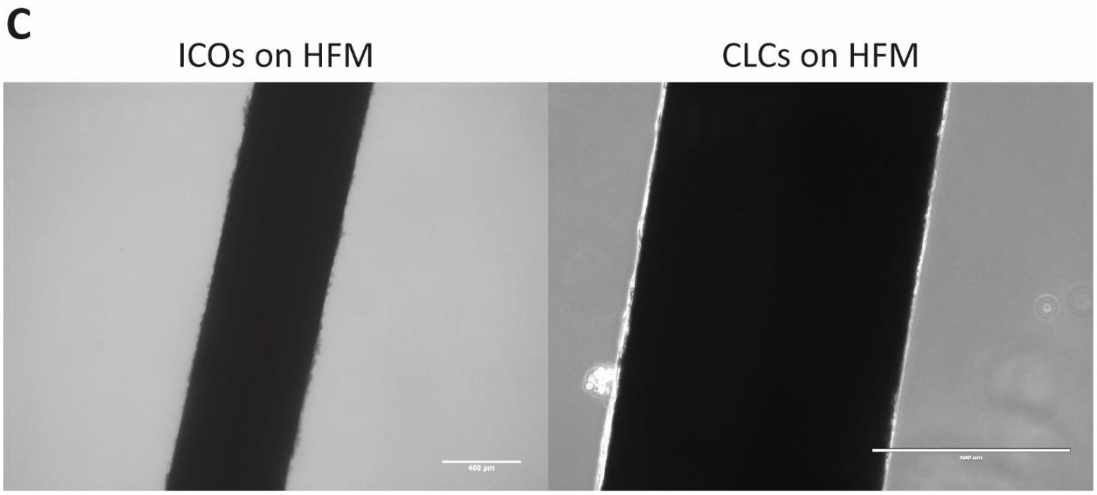

Supplementary Figure 2.

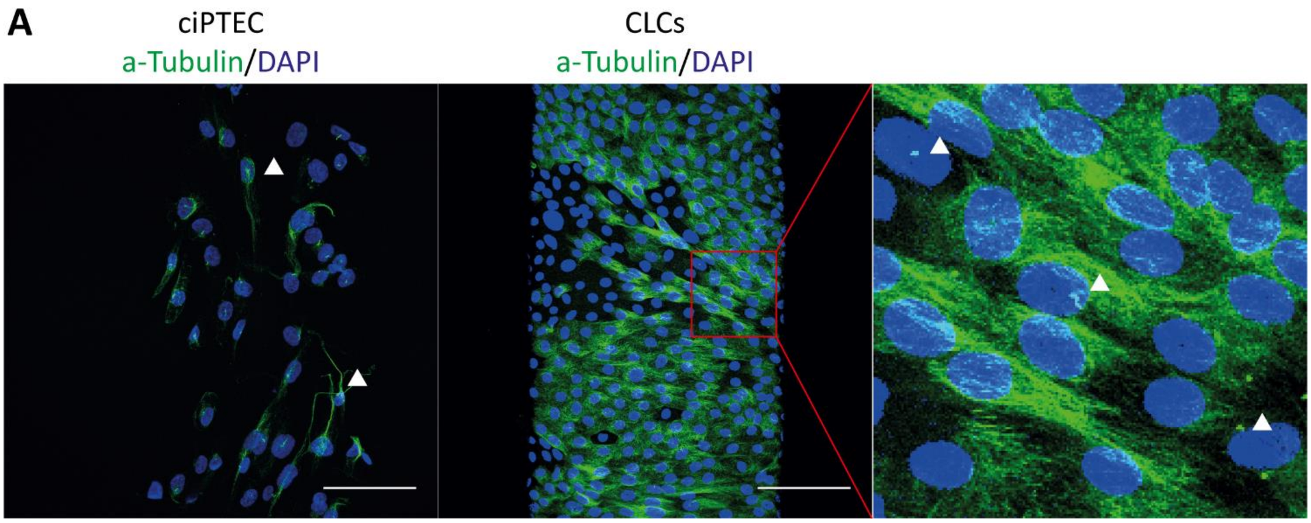

Supplement: Supplementary file 1 [file DataSheet1.PDF]
